# Attention Mechanism and LSTM Network for Fingerprint-Based Indoor Location System

**DOI:** 10.3390/s24051398

**Published:** 2024-02-21

**Authors:** Zhen Wu, Peng Hu, Shuangyue Liu, Tao Pang

**Affiliations:** Department of Mobile Communications and Terminal Research, China Telecom Research Institute, Guangzhou 510000, China; hup11@chinatelecom.cn (P.H.); liusy24@chinatelecom.cn (S.L.); pangtao@chinatelecom.cn (T.P.)

**Keywords:** fingerprinting, indoor localization system, long short-term memory (LSTM), self-attention mechanism

## Abstract

The demand for precise indoor localization services is steadily increasing. Among various methods, fingerprint-based indoor localization has become a popular choice due to its exceptional accuracy, cost-effectiveness, and ease of implementation. However, its performance degrades significantly as a result of multipath signal attenuation and environmental changes. In this paper, we propose an indoor localization method based on fingerprints using self-attention and long short-term memory (LSTM). By integrating a self-attention mechanism and LSTM network, the proposed method exhibits outstanding positioning accuracy and robustness in diverse experimental environments. The performance of the proposed method is evaluated under two different experimental scenarios, which involve 2D and 3D moving trajectories, respectively. The experimental results demonstrate that our approach achieves an average localization error of 1.76 m and 2.83 m in the respective scenarios, outperforming the existing state-of-the-art methods by 42.67% and 31.64%.

## 1. Introduction

The rapid development of global digitization has created a high demand for location-based services (LBS) in many industries [1]. These services have become essential for various systems and applications, including transportation [2], logistics [3,4], emergency response [5], etc. [6,7]. In outdoor environments, mobile users already have access to established outdoor positioning technologies such as the Global Positioning System (GPS) [8] and the BeiDou Satellite Navigation System (BDS) [9] to obtain accurate location information. However, the effectiveness of these technologies is often limited in indoor environments due to the scattering and attenuation effects of satellite signals.

**Figure 1:**
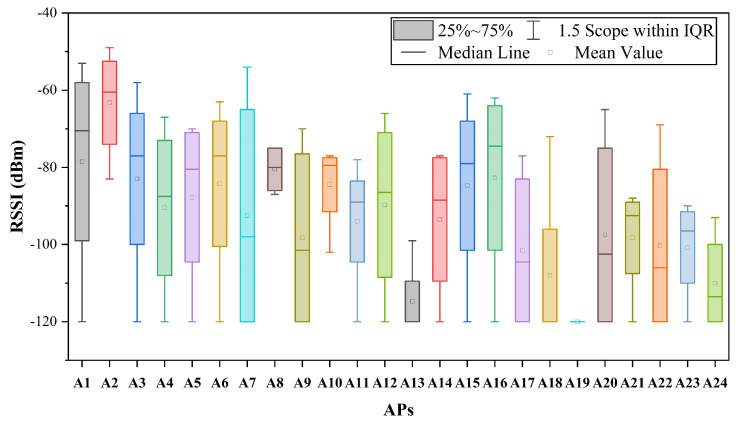
The range of variation in RSSI for APs observed at a fixed location.

In the field of indoor localization, various wireless signals have been proposed and utilized, including Wi-Fi [10,11,12,13], Bluetooth [14,15], ultra-wide bandwidth (UWB) [16,17], radio frequency identification (RFID) [18], and custom radios [19]. Typical ranging-based methods for processing wireless signals in indoor localization involve using information such as angle of arrival (AOA) or time of arrival (TOA) to estimate the specific positions of the user equipment (UE) [20]. However, these methods require prior knowledge of the locations of access points (APs) and are susceptible to errors in the distance measurement between the UE and APs, which can negatively impact the accuracy of the positioning. In contrast to these methods, the fingerprint-based indoor localization method is characterized by simplicity and efficiency [21]. This technique relies on the unique characteristics of wireless signals in indoor environments to create a map or “fingerprint” of the received signal strength indicator (RSSI) at different locations. The fingerprint can then be used to estimate the position of the UE based on the signal strengths measured at that location. Fingerprint-based methods are highly accurate and can offer sub-meter-level positioning accuracy in many cases, making them a promising alternative to ranging-based methods. However, in the context of fingerprint-based methods, the radio propagation environment introduces multi-path effects, shadowing, signal fading, and other forms of signal degradation and distortion, leading to significant fluctuations in RSSI values. In the experiments described in this paper, the observed RSSI values for different APs at a fixed location exhibit a wide range of fluctuations, as illustrated in Figure 1. The fluctuation in RSSI makes it challenging to discern the pattern of RSSI between the test points (TPs) and reference points (RPs), thereby significantly impacting the accuracy of positioning.

With the development of machine learning algorithms in recent decades, numerous machine learning algorithms have been proven to be effective in recognizing the RSSI pattern [22]. M. Brunato et al. proposed applying support vector machines (SVMs) in location fingerprint positioning systems [23]. Hoang et al. introduced a soft range-limited k-nearest neighbors (KNN) fingerprinting algorithm that addresses spatial ambiguity in localization by scaling the fingerprint distance with a range factor based on the physical distance between the previous position of users and the reference location in the database [24]. Fang et al. utilized feedforward neural networks (FNNs) to extract fingerprint features from the RSSI, enabling the accurate localization of the actual position [25]. However, the performance of these algorithms can easily be limited when learning features in complex indoor environments. To achieve superior performance, some research studies have suggested using long short-term memory (LSTM) for handling sequential trajectory prediction in indoor localization systems [10,26,27], which has been experimentally demonstrated to be more effective than the conventional KNN method. Meanwhile, self-attention has been proposed as a promising technique for enhancing the performance of sequence processing tasks [28,29,30,31]. By enabling the model to attend to various regions of the input sequence, self-attention improves its capacity to capture the connections between various features in a sequence.

This paper introduces a novel method named self-attention and LSTM (SA-LSTM) that effectively improves the positioning accuracy and robustness. We conducted experiments in two different scenarios to validate the effectiveness and robustness of the proposed approach. The experimental results demonstrate that SA-LSTM exhibits greater robustness and higher accuracy in indoor localization compared to some of the most advanced algorithms.

The main contributions of this paper are as follows:

1. We propose a novel deep neural network that integrates the self-attention mechanism and LSTM networks for indoor localization. The proposed SA-LSTM method processes the RSSI values of consecutive time instances and predicts the position at the final moment in the input sequence. To the best of our knowledge, this is the first time that the self-attention mechanism and LSTM networks have been fused for RSSI-based fingerprinting localization.
2. Based on LSTM, the SA-LSTM introduces the self-attention mechanism, which enables the LSTM to effectively capture the interdependencies between the RSSI values at different time instances, thereby facilitating the improved extraction of location information and reducing the localization error.
3. We conducted a comparative analysis between our proposed model and several state-of-the-art methods. The experimental results reveal that our proposed SA-LSTM model achieves the highest localization accuracy in both experimental scenarios, demonstrating its robustness and precision.

The rest of this paper is structured as follows. Section 2 provides an overview of related works in the area of fingerprint indoor localization systems. Section 3 presents the technical details of our proposed model. Section 4 outlines the experimental setup utilized in our study. Section 5 presents and analyzes the experimental results obtained from various datasets. Finally, Section 6 offers concluding remarks and outlines our future research plans.

## 2. Related Work

In this section, we present an overview of the existing research on fingerprint-based indoor localization and the application of self-attention mechanisms.

In the current landscape, numerous clustering-based and ensemble-based models have been applied in the field of fingerprint-based indoor localization. In terms of clustering-based models, Bahl et al. [32] were the first to propose the utilization of the KNN algorithm in fingerprint-based indoor localization. By evaluating the Euclidean distance of the RSSI vector from multiple base stations, the KNN algorithm assigns the nearest reference points for target points and computes the average position as their predicted positions. Expanding on KNN, Brunato et al. [23] introduced weighted KNN (WKNN), which calculates the weighted average of reference point positions and enhances the overall positioning accuracy. Within the realm of ensemble-based models, Jedari E. et al. [33] employed a random forest classifier for RSSI-based indoor positioning. Experimental outcomes demonstrate that the random forest classifier outperforms KNN in terms of positioning accuracy. Furthermore, the effectiveness of the AdaBoost method is validated in [34], where AdaBoost is utilized to leverage the channel state information (CSI) from Wi-Fi signals for localization. In another study, Singh N. et al. [35] presented an indoor localization scheme based on XGBoost, capable of accurately classifying the positions of mobile devices in indoor environments, achieving an average positioning error of 4.93 m, 7.02 m, and 1.5 m in three different environments. Moreover, Tekler Z. D. et al. [36] proposed a supervised ensemble model and a semi-supervised clustering model and evaluation revealed that the supervised ensemble model outperforms in terms of positioning accuracy.

Except for ensemble-based models, Yerbolat Khassanov et al. explored the use of end-to-end sequence models for Wi-Fi-based indoor localization at a finer level [10]. The study showed that the localization task can be effectively formulated as a sequence learning problem using recurrent neural networks (RNNs) with regression output. The use of regression output allows for estimating three-dimensional positions and enables scalability to larger areas. The experiments conducted on the Wi-Fi dataset reveal that RNN models outperform non-sequential models such as KNN and FNN, achieving an average positioning error of 3.05 m for finer-level localization tasks. Furthermore, Zhenghua Chen et al. proposed a deep LSTM network for indoor localization using Wi-Fi fingerprinting [37]. The network incorporates a local feature extractor that enables the encoding of temporal dependencies and the learning of high-level representations based on the extracted sequential local features. The experimental results demonstrate that the proposed approach achieves state-of-the-art localization performance, with mean localization errors of 1.48 m and 1.75 m in research lab and office environments, respectively.

In the field of neural machine translation tasks, Bahdanau et al. introduced the self-attention mechanism to the encoder–decoder model. This enables the model to learn alignment and translation simultaneously, allowing for the adaptive selection of encoded vectors [38]. Building on the effectiveness of the self-attention mechanism, several other deep learning architectures have been redesigned to incorporate self-attention for performance enhancement. Yang C. H. et al. [39] integrated self-attention into DNN to effectively improve the adversarial speech signals. Additionally, Mittag G. et al. [40] proposed a deep CNN-self-attention model for multidimensional speech quality prediction, which outperformed CNN. Moreover, an LSTM structure based on the self-attention mechanism was introduced in [41], which showed a superior performance in forecasting temporal sequences compared to other benchmark methods.

In general, LSTM has demonstrated exceptional performance in sequence prediction tasks, including fingerprint localization. It has been experimentally verified that it outperforms conventional methods such as KNN and WKNN. Additionally, the self-attention mechanism enables the model to consider the relationship between each element in the sequence. This leads to a better understanding of contextual information and a more precise processing of sequence data. Based upon that, we propose an SA-LSTM model with high accuracy and strong robustness for indoor localization systems based on fingerprinting.

## 3. Methodology

In this section, we will begin by introducing the framework of the SA-LSTM-based localization algorithm. Subsequently, we will provide detailed introductions to the working principles of its subcomponents.

### 3.1. SA-LSTM-Based Localization Algorithm

**Figure 2:**
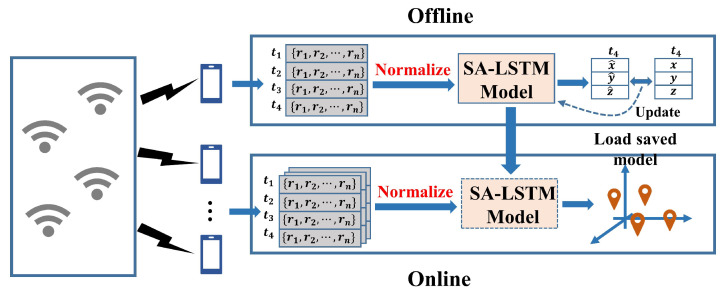
The framework of SA-LSTM-based localization algorithm. $t_{i}$: the *i*th time slice. $r_{i}$: the RSSI value from AP *i*. $\left(\hat{x},\hat{y},\hat{z}\right)$: the coordinates of predicted positions. (x,y,z): the coordinates of real positions.

Figure 2 illustrates the framework of the SA-LSTM-based localization algorithm, comprising an offline training stage and an online estimation stage. During the offline training stage, the RSSI values collected at different points and their corresponding coordinates of locations are recorded and stored in the fingerprint database. Subsequently, the collected RSSI data are normalized and used to train the SA-LSTM network. The trainable weights of the SA-LSTM network will be updated to minimize the loss between the output and the ground true locations. The trainable weights of the SA-LSTM network are adjusted to minimize the loss between the output and the actual locations. During the online estimation stage, real-time RSSI data from the device are normalized and input into the trained SA-LSTM model, which then generates real-time location estimates.

### 3.2. LSTM Network

LSTM is a unique form of recurrent neural network that has been extensively researched in deep learning. In contrast to conventional RNN, the LSTM network introduces gated states to modulate the flow of information, thereby enabling it to selectively retain relevant information over extended periods while filtering out irrelevant data, which allows it to effectively analyze the long temporal sequences.

**Figure 3:**
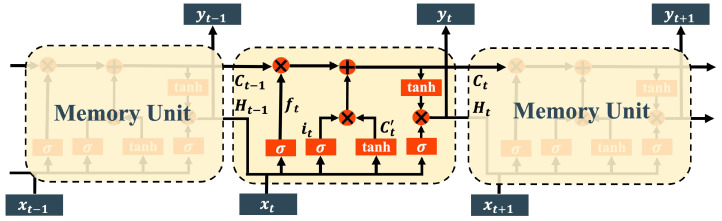
Architecture of LSTM.

Figure 3 shows the common architecture of LSTM, which is composed of connected memory units. In this context, $C_{t}$ and $H_{t}$ represent the unit state and hidden state at time *t*, respectively. Focus on the time *t*, the memory unit receives the $C_{t-1}$ and $H_{t-1}$ from the previous memory unit, as well as the current input value $x_{t}$. After performing internal arithmetic operations, the unit generates the updated cell state $C_{t}$ and hidden state $H_{t}$, which are subsequently passed on to the next memory unit. The hidden state $H_{t}$ also serves as the output result $y_{t}$ corresponding to the current time step.

Each memory unit in the LSTM architecture comprises three components: a forget gate, an input gate, and an output gate. The forget gate can be mathematically expressed as follows:

(1) $$f_{t}=\sigma \left(W_{f}\left[H_{t-1},x_{t}\right]+b_{f}\right)$$

Here, σ represents the activation function, while $W_{f}$ and $b_{f}$ denote the weights and bias of the forget gate, respectively. By multiplying with $C_{t-1}$, the forget gate aims to decide what information should be forgotten in it. For the implementation of the input gate, the sigmoid activation function [42] is initially employed to determine the values that require updating, as illustrated in (2), where $W_{i}$ and $b_{i}$ are the weight matrices and the bias. Subsequently, the tanh activation function generates a new candidate value, denoted by $C_{t}^{\prime }$. The mathematical expression is shown in (3), where $W_{c}$ and $b_{c}$ represent the weight matrices and the bias, respectively.

(2) $$i_{t}=\sigma \left(W_{i}\left[H_{t-1},x_{t}\right]+b_{i}\right)$$

(3) $$C_{t}^{\prime }=tanh\left(W_{c}\left[H_{t-1},x_{t}\right]+b_{c}\right)$$

These two stages are subsequently combined to generate an updated state value, which is then added to the unit state to update the long-term memory of LSTM (i.e., $C_{t}$), as indicated by the following equation:

(4) $$C_{t}=f_{t}⊙C_{t-1}+i_{t}⊙C_{t}^{\prime }$$

⊙ represents the Hadamard product operation. The output gate is responsible for generating the hidden state, which can be calculated as:

(5) $$H_{t}=\sigma \left(W_{o}\left[H_{t-1},x_{t}\right]+b_{o}\right)⊙tanh\left(C_{t}\right)$$

where $W_{o}$ and $b_{o}$ are the weight matrix and the bias of the output gate. LSTM is capable of selectively memorizing and forgetting features via the regulation of three gates, thereby mitigating the issue of long-term dependency. Additionally, LSTM addresses the issue of vanishing gradients that often occurs in RNN. As a result, LSTM has gained widespread adoption in time series prediction tasks.

### 3.3. Self-Attention Mechanism

The attention mechanism is inspired by the human visual attention mechanism, which selectively focuses on specific regions of interest and allocates more attentional resources to extract relevant information while suppressing irrelevant information. Self-attention is a type of attention mechanism, which enables the model to capture the degree of association between each position in a sequence and all other positions. By computing the attention weight of each position with respect to all other positions, the model is able to selectively focus on the most relevant parts of the input sequence and generate more precise predictions or representations.

**Figure 4:**
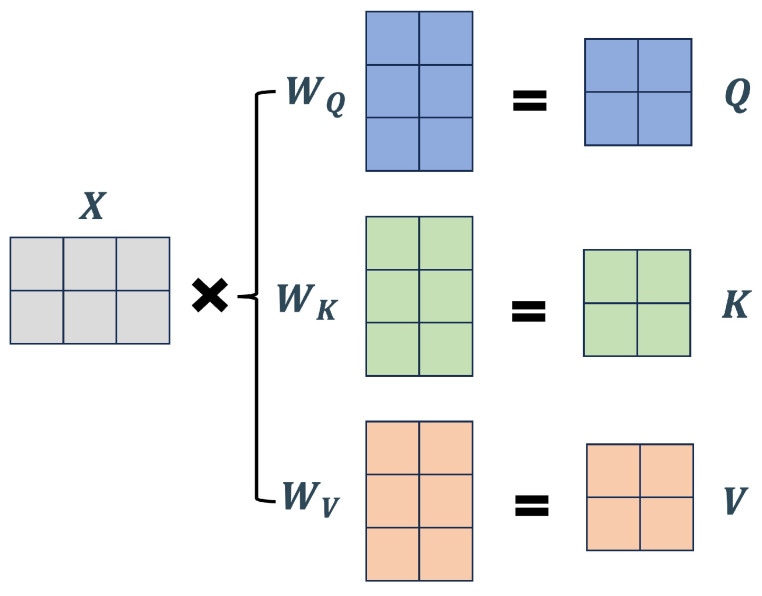
The generation of the Q, K, and V matrices.

The self-attention mechanism is based on the query matrix Q, the key matrix K, and the value matrix V, the generation of which is depicted in Figure 4. Given an input sequence X, the attention mechanism employs three trainable weight matrices (corresponding to $W_{Q}$, $W_{K}$, and $W_{V}$ in Figure 4) to compute the query matrix, the key matrix, and the value matrix V, respectively. By computing the dot product between Q and K, and normalizing the resulting scores using a softmax function, the attention weight coefficients can be obtained, which can be expressed as:

(6) $$AW\left(Q,K\right)=softmax\left(\frac{{\mathrm{QK}}^{T}}{\sqrt{d}}\right)$$

where *d* refers to the dimension of the hidden layer in the key and query matrices. Due to the potentially large dot product of the query matrix Q and the key matrix K when their dimensions are high, numerical instability may occur during training. To address this issue, dividing the dot product by $\sqrt{d}$ normalizes the scale of the product across all dimensions, enhancing the stability and performance of the model. Furthermore, based on the attention weight AW(Q,K), the attention value can be expressed as:

(7) $$A\left(Q,K,V\right)=softmax\left(\frac{{\mathrm{QK}}^{T}}{\sqrt{d}}\right)V$$

**Figure 5:**
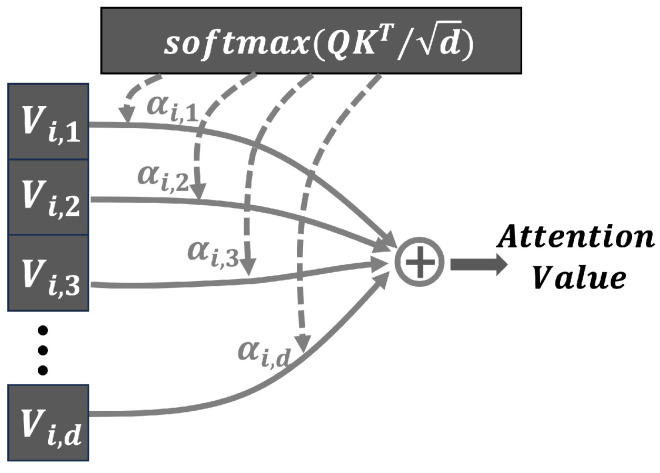
The generation of the attention value.

Specifically, for each position in the sequence, the corresponding value vector is multiplied by its attention weight coefficient. The resulting products are then summed to obtain the attention value, allowing the model to place greater emphasis on the most relevant positions. This process is illustrated in Figure 5, where $\left\{\alpha_{i,1},\alpha_{i,2},\cdots ,\alpha_{i,d}\right\}$ represents the attention weight coefficients.

### 3.4. Proposed SA-LSTM Network

**Figure 6:**
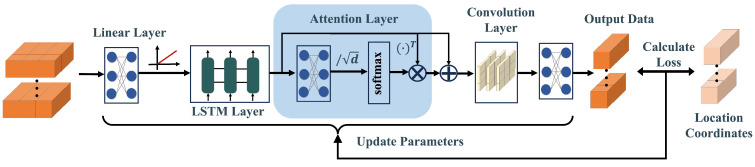
The framework of proposed SA-LSTM model.

Based on the LSTM model and the self-attention mechanism, this paper proposes an SA-LSTM model for indoor localization enhancement. The framework of the SA-LSTM model is depicted in Figure 6. The input data for SA-LSTM are constructed using the collected RSSI data.

#### 3.4.1. Input Sequence Data

At first, a recorded trajectory can be expressed as a matrix:

(8) $$R=\left[\begin{array}{cccc} r_{1,1} & r_{1,2} & \cdots & r_{1,N}\\ r_{2,1} & r_{2,2} & \cdots & r_{2,N}\\ \vdots & \vdots & \ddots & \vdots \\ r_{T,1} & r_{T,2} & \cdots & r_{T,N} \end{array}\right]$$

In this context, *N* refers to the total number of APs, while *T* represents the length of a trajectory. Each element in the matrix R corresponds to the received RSSI. To prepare the data for analysis, we apply the normalization method described in [43]. This involves using the following expression:

(9) $$r_{i,j}^{\prime }={\left(\frac{r_{i,j}-c}{-c}\right)}^{e}$$

where *e* represents the Euler’s number [44]. The constant value *c* should be set to a number less than or equal to the minimum value of RSSI. This ensures that all RSSI values can be scaled between 0 and 1 through normalization. Once the normalization is complete, trajectory segmentation will be performed on all the collected trajectories. Considering trajectories as $\left[\left({\tilde{r}}_{1},l_{1}\right),\left({\tilde{r}}_{2},l_{2}\right),\cdots ,\left({\tilde{r}}_{T},l_{T}\right)\right]$, where ${\tilde{r}}_{i}=\left[r_{1,1},r_{1,2},\cdots ,r_{1,N}\right]$ represents the RSSI from all APs in a given position, while $l_{i}=\left[x_{i},y_{i}\right]$ represents the corresponding coordinates of this position. To facilitate the analysis, each trajectory is divided into smaller segments using a sliding window of a fixed length, denoted by *L*. These segments are then used as inputs for the SA-LSTM model. Mathematically, this process can be expressed as follows:

(10) $$l_{i+L}=F\left({\tilde{r}}_{i},{\tilde{r}}_{i+1},\cdots ,{\tilde{r}}_{i+L-1}\right)$$

where F(·) is the mathematical expression of SA-LSTM, and the $l_{i+L}$ represents the position of the last time step for the input data.

#### 3.4.2. The Layers of Network

After preprocessing the data, the prepared dataset will be fed into the SA-LSTM model. The input layer of the SA-LSTM model employs a fully connected layer with a rectified linear (ReLU) activation function to increase the dimension of the feature space. Mathematically, this can be expressed as follows:

(11) $$X^{\prime }=ReLU\left(W_{1}X+b_{1}\right)$$

The resulting output will then be passed through an LSTM layer to generate the corresponding output for each time step. This output will serve as the input for the subsequent self-attention layer. Within the self-attention layer, several enhancements are implemented to decrease the number of network parameters. As shown in Equation (6), the attention weights are computed using the query matrix Q and the key matrix K. This computation can be further simplified as follows:

(12) $$\begin{array}{cc} AW(X,W_{a}) & =softmax\left(\frac{{\mathrm{QK}}^{T}}{\sqrt{d}}\right)\\ \; & =softmax\left(\frac{\left({\mathrm{XW}}_{Q}\right){\left({\mathrm{XW}}_{K}\right)}^{T}}{\sqrt{d}}\right)\\ \; & =softmax\left(\frac{X\left(W_{Q}{W_{K}}^{T}\right)X^{T}}{\sqrt{d}}\right) \end{array}$$

Given the relationship $W_{A}=W_{Q}{W_{K}}^{T}$, it follows that a fully connected layer with trainable weights $W_{A}$ can be utilized in the attention layer to facilitate the computation of attention weights. Afterward, the output of the fully connected layer will be divided by $\sqrt{d}$ and normalized using the softmax function to obtain the attention weights. It is noteworthy that the output of the LSTM layer contains the information required for SA-LSTM, which means it can be directly considered as the key matrix K. After calculating the attention weights, the next step involves performing a dot product operation between the attention weights and the transposed output from the LSTM layer.

SA-LSTM utilizes a shortcut connection [45] to propagate the attention values obtained from the attention layer, which enhances the backpropagation of gradients and mitigates gradient vanishing. A convolutional layer is then applied to modify the data channels before moving on to the final layer. In the final layer, a fully connected layer is employed to convert the input into location coordinates. The model then calculates the mean square error (MSE) between the predicted output $\tilde{Y}$ and the practical location coordinates Y. The loss is calculated as:

(13) $$\begin{array}{c} L_{\mathrm{MSE}}\left(\tilde{Y},Y\right)=\frac{\sum_{i=1}^{n}(∥Y-\tilde{Y}{∥}^{2})}{n} \end{array}$$

where *n* denotes the number of samples in a batch. According to the loss value, the gradients of the trainable parameters in the model will be computed through backpropagation. Simultaneously, the trainable parameters will be updated in the direction of the negative gradient to minimize the loss value.

## 4. Experimental Setup

To verify the performance of the proposed SA-LSTM method, Bluetooth, and Wi-Fi fingerprint data are applied, which are collected from 2D and 3D moving scenarios, respectively.

### 4.1. Two-Dimensional-Moving Experiment Setup

**Figure 7:**
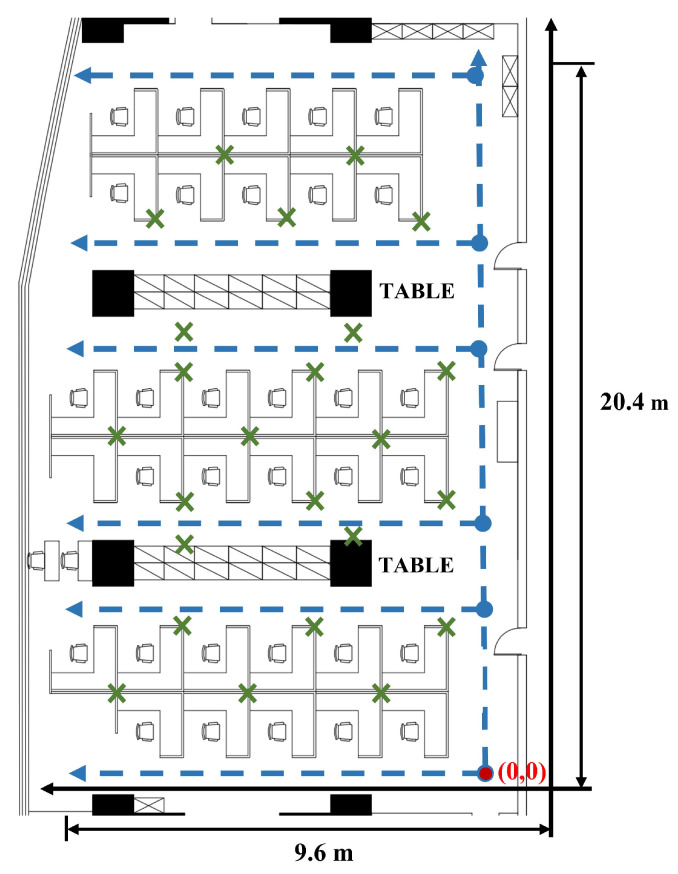
The layout of the office room.

The experimental location for the 2D-moving scenarios is located in an office room on the 28th floor of the Guangdong Telecom Science and Technology Building in China. In this experiment, we deployed 24 Bluetooth beacons at various locations within an office room. These beacons are used to track the movement and location of individuals. Figure 7 shows the layout of the office room, which has an area of 9.6 m × 20.4 m. The solid red dot in Figure 7 represents the origin point in a customized absolute coordinate system. The trajectories used for feature analysis are based on the coordinates of an absolute coordinate system, which serves as a reference point for all position measurements. Additionally, the green cross marks in Figure 7 represent the positions of the Bluetooth beacons, while the blue dashed line indicates the trajectories followed during data collection. The E5 Pilot Positioning Beacon version V006 is applied as the Bluetooth signal transmitter. The specific product parameters are shown in Table 1.

**Figure 8:**
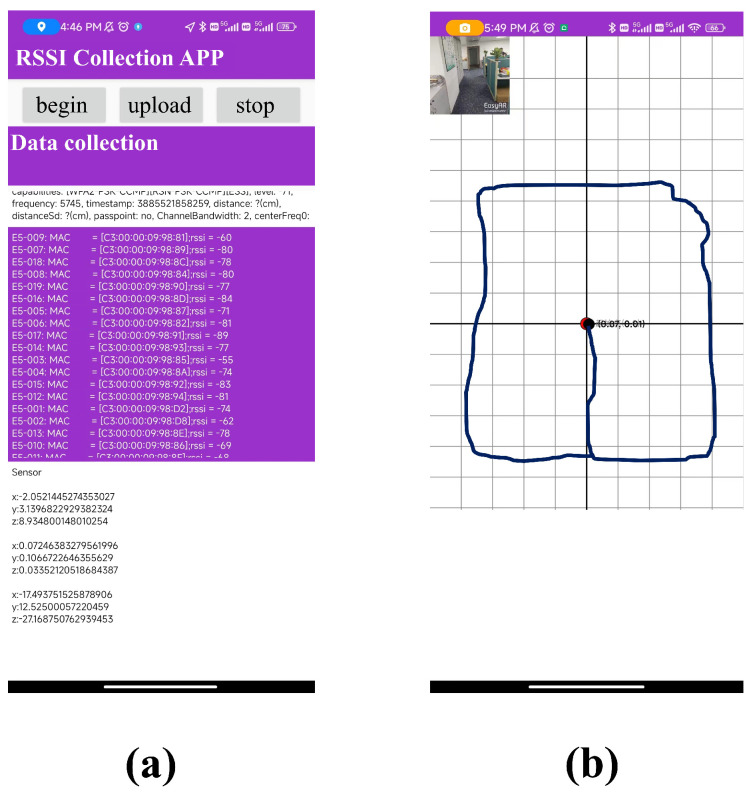
The application used for the (**a**) RSSI collection and (**b**) position recording.

During the experiment, we employed a Xiaomi 10 Pro mobile phone (Xiaomi, Beijing, China) and a ZTE Axon 40 mobile phone (ZTE, Shenzhen, China), both equipped with cameras. To facilitate the data collection task, we developed a mobile phone data collection application capable of capturing Bluetooth signals and logging user positions. In Figure 8, we depict the page of the application. This application leverages the visual simultaneous localization and mapping (VSLAM) framework to acquire real-time coordinates, which were then logged onto files for further analysis. The working principle of VSLAM involves analyzing the visual data captured by the camera to track the movement of the camera and identify features in the environment. By comparing these features with those from previous frames, VSLAM can estimate the motion of the camera and update its position in real time.

To ensure the accuracy of the collected position coordinates, we conducted data acquisition by moving the acquisition device at a constant speed along the predetermined trajectories. The trajectory data for RSSI collection were obtained by following the blue-dashed lines shown in Figure 7. Specifically, we followed each dashed line from the starting point to the end and then retraced our steps from the end back to the exit point, creating two distinct trajectories. The two mobile phones used for data acquisition were programmed to perform signal acquisition and collect corresponding addresses at different times. Overall, these measures ensured that the collected data were of sufficient quality to support our research objectives. The sampling frequency of the collecting devices was set to 1 Hz while moving along the trajectories. In total, we collected 28 trajectories, which were subsequently partitioned into three sets: training, validation, and test sets, in a ratio of 3:1:1. The test and validation datasets mainly contain two categories of trajectories. The first category consists of trajectories that were not included in the training set. The second category includes trajectories that are identical to those in the training set but were collected using different devices.

### 4.2. Three-Dimensional-Moving Experiment Setup

The 3D-moving experiment dataset is publicly available as an open source dataset [10]. In contrast to the 2D-moving experiment, the 3D-moving experiment dataset is based on Wi-Fi fingerprints and covers trajectories across the fourth, fifth, and sixth floors of the C4 building at Nazarbayev University. This dataset provides a comprehensive and representative set of data, enabling a thorough evaluation of the performance of indoor localization systems in complex, multi-floor environments. This Wi-Fi dataset comprises 290 trajectories that were sequentially collected with a fine spatiotemporal resolution. The dataset covers a total area of over 9564 m^2^ across three floors. The experimental environment is equipped with 439 wireless access points. During the experiment, the validation and test trajectories were collected a few days after obtaining the training set. These trajectories were uniquely designed to be dissimilar from the training trajectories. Moreover, the users were authorized to switch floors using the four elevators installed in the building while collecting the data, which helps to evaluate the performance of the model in 3D-moving scenarios. A total of 170 unique trajectories were collected, with an even distribution between the validation and test sets.

### 4.3. SA-LSTM Training Setup

In the two experimental scenarios, the hyperparameters of the SA-LSTM model were adjusted differently. The details of these hyperparameters are presented in Table 2. For each *L* of consecutive input RSSI vectors at a given moment, the network predicts the exact location of the last recorded time point. The initial learning rate is set to 0.001 for both scenarios. During the training process, we reduce the learning rate to one-tenth of the previous rate after a fixed number of training epochs. In the 2D scenario, the learning rate was adjusted every 30 epochs, while in the 3D scenario, the learning rate was adjusted every 20 epochs. All models were trained using an NVIDIA GeForce RTX 2080 Ti GPU, manufactured by NVIDIA, based in Santa Clara, CA, United States.

## 5. Results and Discussion

**Figure 9:**
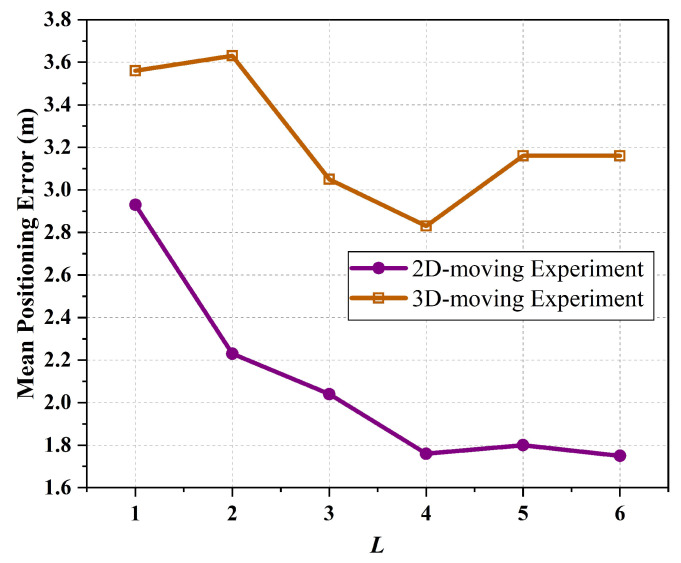
The length of the sliding window against the mean positioning error.

Before comparing the performance of various methods, the sliding window length *L* for the SA-LSTM method needs to be determined. Figure 9 illustrates the mean positioning error as a function of the window size. As shown in the figure, SA-LSTM performs poorly when *L* is set to 1 or 2. As *L* increases, the average localization error of SA-LSTM shows a significant decrease. This occurs because when *L* is set to a smaller value, the network model obtains less information, resulting in lower positioning accuracy. When *L* is taken to 5 or 6, the average localization error fluctuates within a small range. To avoid additional computational complexity, *L* is determined to be set to 4.

To compare our indoor localization approach, we implemented an indoor localization system network based on LSTM, as described in [37]. Additionally, we implemented other methods such as RNN [10], KNN, WKNN, FNN, and linear regression. We adjusted the parameters of these models within a certain range to optimize their performance. During the training process, all the model was validated using the validation set after each training epoch, and the model with the minimum average position error was saved for further evaluation.

The average and maximum positioning errors of all these methods are presented in Table 3. The SA-LSTM method outperforms other methods in terms of average positioning accuracy. Among these methods, the LSTM approach achieves the second-best performance in mean positioning accuracy, following the proposed SA-LSTM method. On the test set, the LSTM method results in a maximum error of 13.73 m and an average error of 3.07 m, which is 0.98 m and 1.31 m higher than the proposed SA-LSTM method. Compared to the RNN method, which has a mean positioning error of 4.16 m and a maximum error of 12.64 m, SA-LSTM improves the positioning accuracy by 2.4 m and 0.29 m. Moreover, SA-LSTM achieves a maximum improvement of 66.85% in average positioning accuracy compared to the linear regression method.

**Figure 10:**
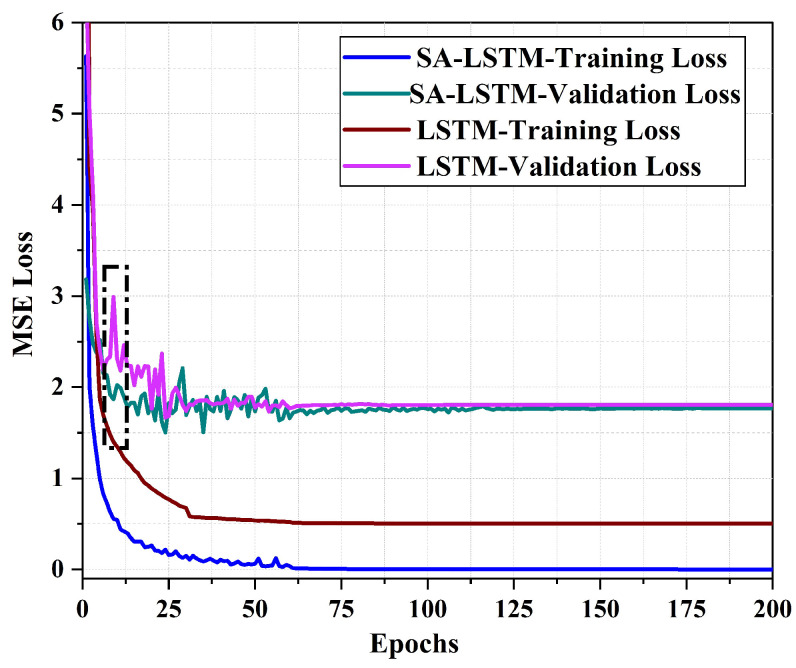
The MSE loss curve of SA-LSTM and LSTM methods in 2D-moving experiment.

Figure 10 illustrates the MSE loss curve of the SA-LSTM and LSTM methods during the training process with 2D-moving trajectories. Our results indicate that exhibits a faster convergence rate in terms of training loss compared to the LSTM model. Moreover, after 200 epochs of training, the training loss of SA-LSTM converges to around 0, while the training loss of LSTM converges to around 0.5. The validation loss of SA-LSTM converges faster to near-stabilization values compared to LSTM, as demonstrated in the black-dotted box in Figure 10. Throughout the entire training process, we observed that the SA-LSTM model achieved a slightly lower minimum validation loss than the LSTM model. These results suggest that the SA-LSTM model is more effective in terms of training efficiency with the help of a self-attention mechanism and shortcut connection.

**Figure 11:**
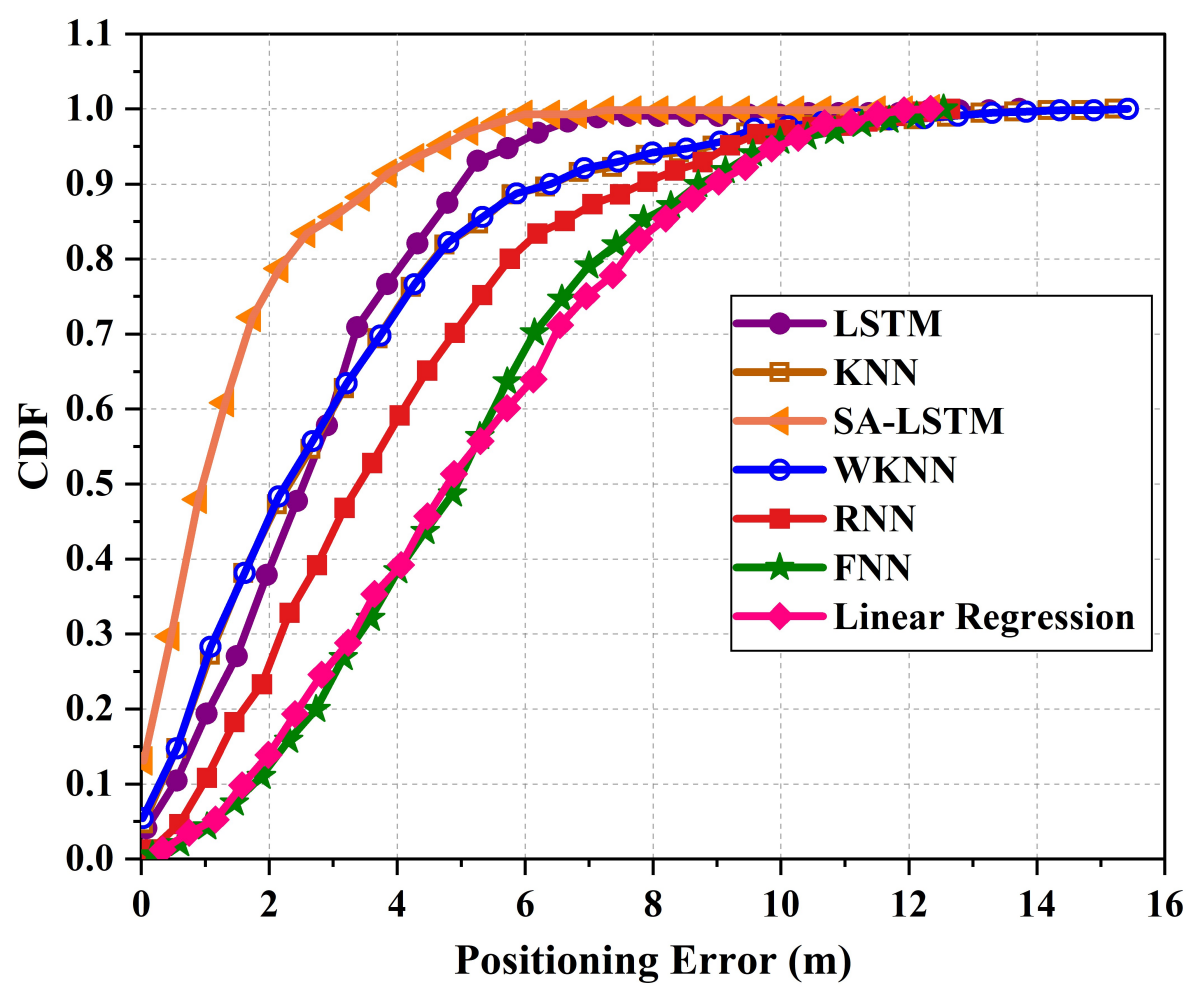
The CDF of localization errors for 2D-moving experiment.

Figure 11 illustrates the cumulative distribution function (CDF) of localization errors for the 2D-moving experiment. In total, a maximum localization error of 12.35 m is recorded for SA-LSTM, 15.22 m is recorded for KNN, and the largest maximum localization error of 15.42 m is recorded for WKNN. Compared to the KNN and WKNN methods, the SA-LSTM method showed a decrease in the maximum localization error by 2.87 m and 3.07 m, respectively. Meanwhile, the maximum localization error of LSTM is 12.47 m, which is also higher than that of SA-LSTM. When considering the 90% percentile of the CDF, the proposed SA-LSTM model demonstrates a 90% location error of approximately under 3.86 m. In comparison, the LSTM, RNN, and KNN models exhibit location errors of around 4.36 m, 5.74 m, and 6.31 m, respectively. This suggests that the proposed SA-LSTM can achieve an improvement of 11.47%, 32.75%, and 63.47% in the 90% CDF compared to LSTM, RNN, and KNN, respectively.

Regarding the 3D-moving experiment, the proposed SA-LSTM model continues to exhibit superior performance in the localization system. Similarly, we compare the average and maximum positioning error of KNN, WKNN, FNN, linear regression, RNN, LSTM, and SA-LSTM. As shown in Table 4, the proposed SA-LSTM achieves an average positioning error of 2.83 m and a maximum positioning error of 57.64 m in the 3D-moving experiment. Compared to LSTM, SA-LSTM improves the average positioning accuracy by 31.64%. In addition, SA-LSTM reduces the average positioning errors by 2.1 m and the maximum localization errors by 3.32 m compared to RNN. Compared to KNN and WKNN, the SA-LSTM has an average positioning error that is 0.62 m and 0.61 m lower, respectively. The SA-LSTM has achieved the lowest average positioning error and the maximum positioning error in scenes involving 3D motion.

**Figure 12:**
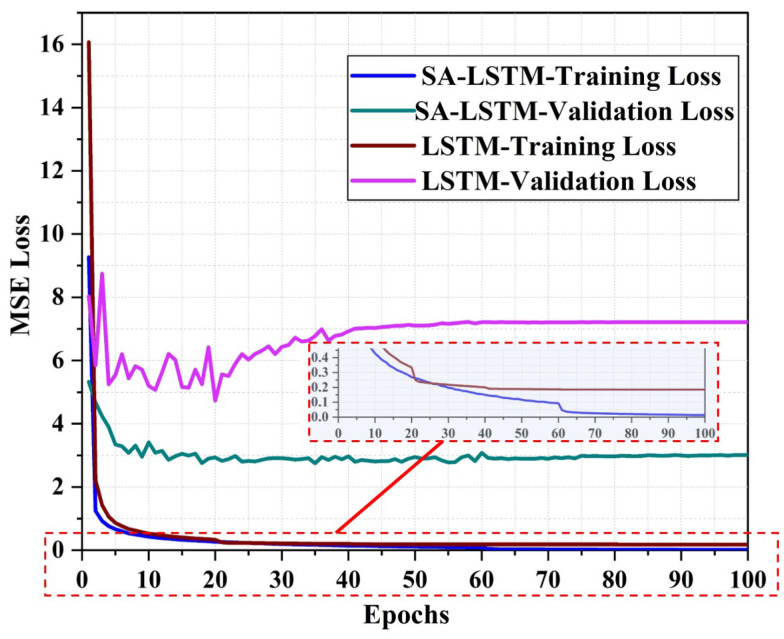
The MSE loss curve of SA-LSTM and LSTM methods in 3D-moving experiment.

The loss curves for SA-LSTM and LSTM in the 3D-moving experiment are depicted in Figure 12. The training loss of SA-LSTM and LSTM converge at a similar rate. As shown in the zoomed-in image in Figure 12, the final convergence value of SA-LSTM is a bit lower. In terms of the validation loss, the SA-LSTM model exhibited a better performance than the LSTM model. Specifically, the validation loss of SA-LSTM could eventually converge to 3, while that of LSTM remained above 4. Based on these findings, we can conclude that our proposed SA-LSTM model is significantly more efficient in terms of training efficiency compared to the conventional LSTM model.

**Figure 13:**
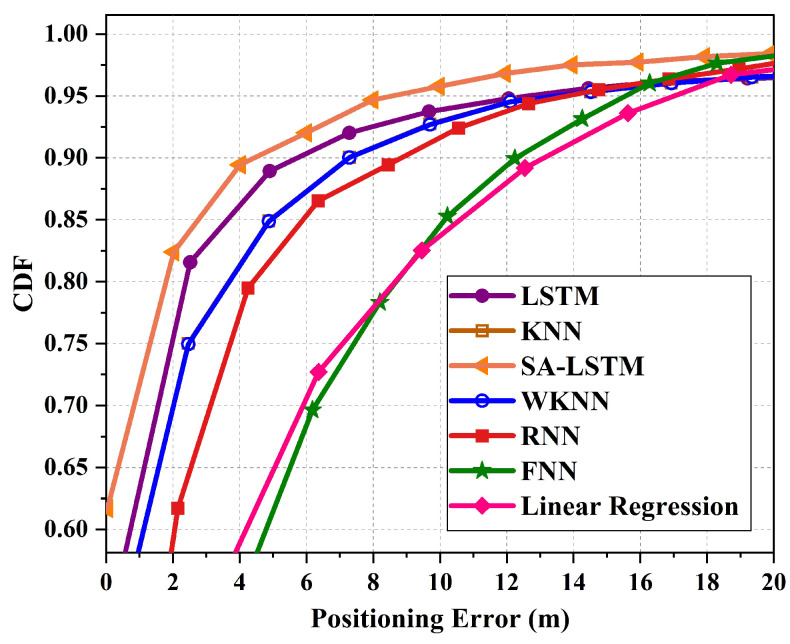
The CDF of localization errors for 3D-moving experiment.

Figure 13 illustrates the CDF of localization errors for the 3D-moving experiment. Overall, the proposed SA-LSTM still outperforms the other classical algorithms. The LSTM network performs the second best, which achieves a 90% location error below 6 m, while RNN achieves a 90% location error below 8.45 m. Compared to LSTM and RNN, SA-LSTM decreased the 90% CDF by 1.99 m and 4.44 m.

**Figure 14:**
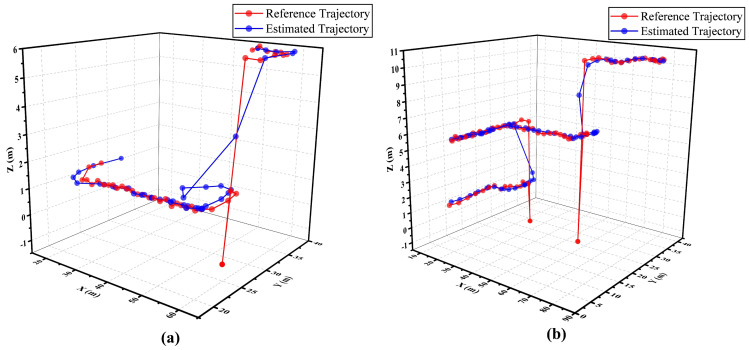
Schematic diagram of referenced and estimated trajectories with a range of movement involving (**a**) two floors and (**b**) three floors.

Furthermore, a couple of estimated trajectories are drawn in a 3D-moving experiment using the SA-LSTM model. Figure 14a,b depict the moving trajectories, which involve transitions between two and three different floors, respectively. The red lines correspond to the reference trajectory, whereas the blue lines depict the estimated trajectories generated by SA-LSTM. The experimental results indicate that the measured position points in the referenced trajectories exhibit anomalous behavior during pedestrian transitions between different floors. This behavior is attributed to the reliance on elevators for inter-floor movement, which leads to abnormal fluctuations in the measurement signal, resulting in anomalous measured positions. From the trajectories shown in Figure 14a,b, it can be demonstrated that the proposed SA-LSTM model exhibits a satisfactory performance when the pedestrians under test move within a single floor. However, when pedestrians move between floors, the estimated position points generated by the SA-LSTM model may exhibit some fluctuations within a narrow range. Nevertheless, once the pedestrians reach a specific floor, the SA-LSTM model can promptly resume its effective operation.

**Figure 15:**
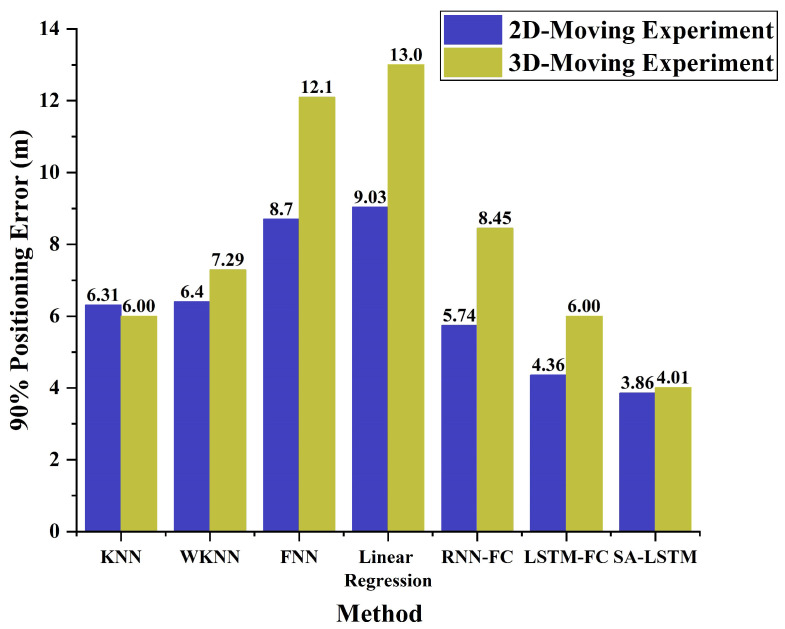
The histogram of a 90% positioning error for two experiments.

The 90% quantile of CDF is an important performance evaluation metric in location systems, as highlighted in 3GPP Rel.18 [46]. To comprehensively evaluate the performance of each algorithm in both 2D-moving and 3D-moving experiments, we calculate the 90% error for each algorithm and present the results in Figure 15.

In both experimental scenarios, SA-LSTM demonstrates the highest localization accuracy compared to the other algorithms, as indicated by its remarkably low 90% positioning error. Under the 3D-moving experimental environment, SA-LSTM achieves a 90% localization error under 3.86 m, which is 0.5 m and 1.88 m lower than that of LSTM and RNN, respectively. Compared to classical KNN algorithms, the SA-LSTM model consistently exhibits a lower 90% positioning error under both experimental environments. These results suggest that SA-LSTM demonstrates high accuracy and stability in the field of indoor positioning, highlighting its potential to outperform traditional methods and pave the way for more advanced and reliable indoor positioning systems.

**Figure 16:**
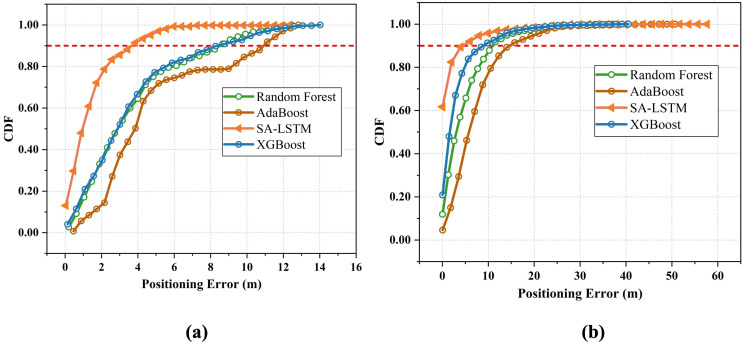
The CDF of SA-LSTM and implemented ensemble-based models in (**a**) 2D-moving experiment and (**b**) 3D-moving experiment.

Furthermore, we implemented several ensemble-based algorithms in the mainstream and compared their performance to that of the proposed SA-LSTM. As depicted in Figure 16, the random forest and AdaBoost exhibited a similar positioning accuracy in the 2D-moving experiment, with an average positioning error of 3.96 m. In the 3D-moving experiment, random forest and AdaBoost demonstrate average positioning errors of 5.69 m and 4.37 m, respectively. Additionally, the SA-LSTM model shows lower positioning errors regarding the 90% CDF in both experimental environments. When compared to the SA-LSTM and LSTM algorithms, the ensemble-based models only focus on the wireless fingerprint signal characteristics at the current moment and do not consider the temporal characteristics of the signal. Moreover, the fluctuation of RSSI can lead to changes in the RSSI pattern at a particular location. These factors seriously impair the performance of these ensemble-based models.

Based on our experimental results, the proposed SA-LSTM shows an outstanding performance in RSSI-based fingerprinting indoor positioning. However, there are still a number of limitations that need to be addressed in our future work. However, there are still several limitations that need to be addressed in our future work. We identified that the density of deployed beacons has a significant impact on the performance of SA-LSTM. To achieve a high positioning accuracy, we tried to have a Bluetooth beacon within every 8 m^2^ based on our beacon configuration. Nevertheless, this strategy necessitates a great number of beacons for large areas. Our future plan involves developing a positioning method that integrates Bluetooth signal data fusion with Wi-Fi and cellular signals. By leveraging these existing wireless signals, we aim to reduce the number of required Bluetooth beacons. Furthermore, we observed that the performance of SA-LSTM is influenced by the movement trajectory. While the training and testing trajectories do not necessarily need to align in the experiments discussed in this paper, it is essential for the training trajectory to comprehensively cover the entire experimental area to ensure localization accuracy. In our future research, we will focus on enhancing the fingerprint acquisition method to mitigate the challenges and costs associated with RSSI acquisition. Finally, due to resource constraints, the performance of SA-LSTM was only validated in two specific environments. As illustrated in the experimental results, SA-LSTM demonstrated a superior performance in an office room compared to the C4 building. This discrepancy can be attributed to the larger size of the C4 building and the increased obstruction by objects within it. Theoretically, the localization accuracy of SA-LSTM is anticipated to be higher in less obstructed environments. For future research endeavors, we aim to validate our approach in a more diverse array of environments.

## 6. Conclusions

This paper introduces a novel SA-LSTM method for fingerprint-based indoor localization systems. The proposed model utilizes the self-attention mechanism to calculate attention scores between each element and all other elements in the output sequence of the LSTM. This enables the SA-LSTM model to focus on the relationship between the position features at different time steps, thereby improving the accuracy of real-time position estimation. The performance of SA-LSTM has been evaluated under various experimental environments that involve 2D and 3D moving trajectories. The experimental results show that SA-LSTM achieves an average localization error of 1.76 m and 2.83 m in the respective scenarios, with 90% of the positioning errors being under 3.86 m and 4.01 m, respectively. Furthermore, when compared with existing state-of-the-art methods in the same test environment, SA-LSTM exhibits a significant improvement in positioning accuracy by 42.67% to 31.64% under the same test environment.

Our study has successfully showcased the potential of the self-attention mechanism in enhancing the accuracy and efficiency of indoor localization systems. In our future work, we plan to conduct further research to explore the applicability and effectiveness of this mechanism in improving the accuracy of indoor localization.

## Figures and Tables

**Table 1:** The product parameters of the Bluetooth beacon.

Parameters	Values
Bluetooth version	BLE 5.0
Bluetooth protocol	iBeacon
Working temperature	−30∼25 °C
Maximum transmission distance	120 m
Transmitted power	−30∼+4 dBm (default: 0 dBm)
Broadcast interval	100 ms∼10 s (default: 500 ms)

**Table 2:** The hyperparameters of SA-LSTM.

Layer	2D Experiment	3D Experiment
Linear layer 1	(24 × 64)	(436 × 128)
LSTM layer	(64 × 64)	(128 × 128)
Linear layer 2	(64 × 4)	(128 × 4)
Convolution layer	3 × 3 kernels, 1 filter	3 × 3 kernels, 1 filter
Linear layer 3	(62 × 2)	(126 × 3)
Batch size	2	2
Initial learning rate	0.001	0.001
Optimizer	Adam	Adam
Loss function	MSE	MSE
Training epochs	200	100

**Table 3:** The positioning error for 2D-moving experiment.

Method	Average Error (m)		Maximum Error (m)	
	**Validation Set**	**Test Set**	**Validation Set**	**Test Set**
KNN	2.53	3.36	18.39	15.22
WKNN	2.53	3.33	18.41	15.42
FNN	3.49	5.28	12.44	12.54
Linear regression	3.64	5.31	13.06	**12.34**
RNN	3.37	4.16	12.67	12.64
LSTM	2.57	3.07	13.73	13.73
SA-LSTM	**1.67**	**1.76**	**12.35**	12.35

**Table 4:** The positioning error for 3D-moving experiment.

Method	Average Error (m)		Maximum Error (m)	
	**Validation Set**	**Test Set**	**Validation Set**	**Test Set**
KNN	3.42	3.45	68.95	69.99
WKNN	3.41	3.44	68.95	69.99
FNN	6.41	6.81	68.79	58.70
Linear regression	7.06	7.56	100.44	89.74
RNN	3.73	4.93	40.71	60.96
LSTM	3.91	4.14	66.91	69.29
SA-LSTM	**2.56**	**2.83**	**28.46**	**57.64**

## Data Availability

Data are contained within the article.

## Abbreviations

The following abbreviations are used in this manuscript:

LBS	Location-based services
GPS	Global Positioning System
BDS	BeiDou Satellite Navigation System
UWB	Ultra-wide bandwidth
RFID	Radio frequency identification
AOA	Angle of arrival
TOA	Time of arrival
APs	Access points
UE	User equipment
LSTM	Long short-term memory
RSSI	Received signal strength indicator
TPs	Test points
RPs	Reference points
KNN	K-Nearest neighbors
WKNN	Weighted K-nearest neighbors
SVM	Support vector machines
FNN	Feedforward neural networks
SA-LSTM	Self-attention and LSTM
VSLAM	Simultaneous localization and mapping
RNN	Recurrent neural networks

## References

[1] Zhong S., Li L., Liu Y.G., Yang Y.R. (2004). Privacy-Preserving Location-Based Services for Mobile Users in Wireless Networks.

[2] Gao L., Xiong L., Xia X., Lu Y., Yu Z., Khajepour A. (2022). Improved vehicle localization using on-board sensors and vehicle lateral velocity. IEEE Sens. J..

[3] Balaji B., Xu J., Nwokafor A., Gupta R., Agarwal Y. Sentinel: Occupancy based HVAC actuation using existing WiFi infrastructure within commercial buildings. Proceedings of the 11th ACM Conference on Embedded Networked Sensor Systems.

[4] Tekler Z.D., Chong A. (2022). Occupancy prediction using deep learning approaches across multiple space types: A minimum sensing strategy. Build. Environ..

[5] Filippoupolitis A., Oliff W., Loukas G. Bluetooth low energy based occupancy detection for emergency management. Proceedings of the 2016 15th International Conference on Ubiquitous Computing and Communications and 2016 International Symposium on Cyberspace and Security (IUCC-CSS).

[6] Nemra A., Aouf N. (2010). Robust INS/GPS sensor fusion for UAV localization using SDRE nonlinear filtering. IEEE Sens. J..

[7] Mulloni A., Seichter H., Schmalstieg D. Handheld augmented reality indoor navigation with activity-based instructions. Proceedings of the 13th International Conference on Human Computer Interaction with Mobile Devices and Services.

[8] Spilker J.J., Axelrad P., Parkinson B.W., Enge P. (1996). Global Positioning System: Theory and Applications.

[9] Yang Y., Gao W., Guo S., Mao Y., Yang Y. (2019). Introduction to BeiDou-3 navigation satellite system. Navigation.

[10] Khassanov Y., Nurpeiissov M., Sarkytbayev A., Kuzdeuov A., Varol H.A. Finer-level sequential wifi-based indoor localization. Proceedings of the 2021 IEEE/SICE International Symposium on System Integration (SII).

[11] Salamah A.H., Tamazin M., Sharkas M.A., Khedr M. An enhanced WiFi indoor localization system based on machine learning. Proceedings of the 2016 International Conference on Indoor Positioning and Indoor Navigation (IPIN).

[12] Abbas M., Elhamshary M., Rizk H., Torki M., Youssef M. WiDeep: WiFi-based accurate and robust indoor localization system using deep learning. Proceedings of the 2019 IEEE International Conference on Pervasive Computing and Communications (PerCom).

[13] Chen C., Chen Y., Lai H.Q., Han Y., Liu K.R. High accuracy indoor localization: A WiFi-based approach. Proceedings of the 2016 IEEE International Conference on Acoustics, Speech and Signal Processing (ICASSP).

[14] Altini M., Brunelli D., Farella E., Benini L. Bluetooth indoor localization with multiple neural networks. Proceedings of the IEEE 5th International Symposium on Wireless Pervasive Computing 2010.

[15] Wang Y., Ye Q., Cheng J., Wang L. RSSI-based bluetooth indoor localization. Proceedings of the 2015 11th International Conference on Mobile Ad-Hoc and Sensor Networks (MSN).

[16] Zhang C., Kuhn M., Merkl B., Fathy A.E., Mahfouz M. Accurate UWB indoor localization system utilizing time difference of arrival approach. Proceedings of the 2006 IEEE Radio and Wireless Symposium.

[17] Poulose A., Han D.S. (2020). UWB indoor localization using deep learning LSTM networks. Appl. Sci..

[18] Montaser A., Moselhi O. (2014). RFID indoor location identification for construction projects. Autom. Constr..

[19] Chen Y., Lymberopoulos D., Liu J., Priyantha B. (2013). Indoor localization using FM signals. IEEE Trans. Mob. Comput..

[20] Lan T., Wang X., Chen Z., Zhu J., Zhang S. (2022). Fingerprint augment based on super-resolution for WiFi fingerprint based indoor localization. IEEE Sens. J..

[21] Torres-Sospedra J., Montoliu R., Martínez-Usó A., Avariento J.P., Arnau T.J., Benedito-Bordonau M., Huerta J. UJIIndoorLoc: A new multi-building and multi-floor database for WLAN fingerprint-based indoor localization problems. Proceedings of the 2014 International Conference on Indoor Positioning and Indoor Navigation (IPIN).

[22] Roy P., Chowdhury C. (2021). A survey of machine learning techniques for indoor localization and navigation systems. J. Intell. Robot. Syst..

[23] Brunato M., Battiti R. (2005). Statistical learning theory for location fingerprinting in wireless LANs. Comput. Netw..

[24] Hoang M.T., Zhu Y., Yuen B., Reese T., Dong X., Lu T., Westendorp R., Xie M. (2018). A soft range limited K-nearest neighbors algorithm for indoor localization enhancement. IEEE Sens. J..

[25] Fang S.H., Lin T.N. (2008). Indoor location system based on discriminant-adaptive neural network in IEEE 802.11 environments. IEEE Trans. Neural Netw..

[26] Nurpeiissov M., Kuzdeuov A., Assylkhanov A., Khassanov Y., Varol H.A. End-to-end sequential indoor localization using smartphone inertial sensors and WiFi. Proceedings of the 2022 IEEE/SICE International Symposium on System Integration (SII).

[27] Zhang Y., Qu C., Wang Y. (2020). An indoor positioning method based on CSI by using features optimization mechanism with LSTM. IEEE Sens. J..

[28] Gibbons F.X. (1990). Self-attention and behavior: A review and theoretical update. Adv. Exp. Soc. Psychol..

[29] Zhao H., Jia J., Koltun V. Exploring self-attention for image recognition. Proceedings of the IEEE/CVF Conference on Computer Vision and Pattern Recognition.

[30] Humphreys G.W., Sui J. (2016). Attentional control and the self: The self-attention network (SAN). Cogn. Neurosci..

[31] Vaswani A., Shazeer N., Parmar N., Uszkoreit J., Jones L., Gomez A.N., Kaiser Ł., Polosukhin I. Attention is all you need. Proceedings of the Advances in Neural Information Processing Systems 30 (NIPS 2017).

[32] Bahl P., Padmanabhan V.N. RADAR: An in-building RF-based user location and tracking system. Proceedings of the Proceedings IEEE INFOCOM 2000, Conference on Computer Communications, Nineteenth Annual Joint Conference of the IEEE Computer and Communications Societies (Cat. No. 00CH37064).

[33] Jedari E., Wu Z., Rashidzadeh R., Saif M. Wi-Fi based indoor location positioning employing random forest classifier. Proceedings of the 2015 International Conference on Indoor Positioning and Indoor Navigation (IPIN).

[34] Ding J., Wang Y., Fu S., Si H., Zhang J., Gao S. (2022). Multiview features fusion and Adaboost based indoor localization on Wifi platform. IEEE Sens. J..

[35] Singh N., Choe S., Punmiya R., Kaur N. (2022). XGBLoc: XGBoost-Based Indoor Localization in Multi-Building Multi-Floor Environments. Sensors.

[36] Tekler Z.D., Low R., Gunay B., Andersen R.K., Blessing L. (2020). A scalable Bluetooth Low Energy approach to identify occupancy patterns and profiles in office spaces. Build. Environ..

[37] Chen Z., Zou H., Yang J., Jiang H., Xie L. (2019). WiFi fingerprinting indoor localization using local feature-based deep LSTM. IEEE Syst. J..

[38] Chorowski J.K., Bahdanau D., Serdyuk D., Cho K., Bengio Y. (2015). Attention-based models for speech recognition. Advances in Neural Information Processing Systems.

[39] Yang C.H., Qi J., Chen P.Y., Ma X., Lee C.H. Characterizing speech adversarial examples using self-attention u-net enhancement. Proceedings of the ICASSP 2020—2020 IEEE International Conference on Acoustics, Speech and Signal Processing (ICASSP).

[40] Mittag G., Naderi B., Chehadi A., Möller S. (2021). Nisqa: A deep cnn-self-attention model for multidimensional speech quality prediction with crowdsourced datasets. arXiv.

[41] Zang H., Xu R., Cheng L., Ding T., Liu L., Wei Z., Sun G. (2021). Residential load forecasting based on LSTM fusing self-attention mechanism with pooling. Energy.

[42] Finney D.J. (1947). Probit analysis: A statistical treatment of the sigmoid response curve. J. R. Stat. Soc..

[43] Torres-Sospedra J., Montoliu R., Trilles S., Belmonte Ó., Huerta J. (2015). Comprehensive analysis of distance and similarity measures for Wi-Fi fingerprinting indoor positioning systems. Expert Syst. Appl..

[44] Song X., Fan X., Xiang C., Ye Q., Liu L., Wang Z., He X., Yang N., Fang G. (2019). A novel convolutional neural network based indoor localization framework with WiFi fingerprinting. IEEE Access.

[45] He K., Zhang X., Ren S., Sun J. Deep residual learning for image recognition. Proceedings of the IEEE Conference on Computer Vision and Pattern Recognition.

[46] Morselli F., Razavi S.M., Win M.Z., Conti A. (2023). Soft information based localization for 5G networks and beyond. IEEE Trans. Wirel. Commun..

